# Comparative Evaluation of Platelet-Rich Fibrin (PRF) and Concentrated Growth Factor (CGF) as Carriers for Antibiotics—In Vitro Study

**DOI:** 10.3390/ijms26094303

**Published:** 2025-05-01

**Authors:** Wojciech Niemczyk, Małgorzata Kępa, Jacek Żurek, Ali Aboud, Dariusz Skaba, Rafał Wiench

**Affiliations:** 1 Department of Periodontal Diseases and Oral Mucosa Diseases, Faculty of Medical Sciences in Zabrze, Medical University of Silesia, Pl. Traugutta 2, 41-800 Zabrze, Poland; 2 Medical Center of Innovation, Wroclaw Medical University, Krakowska 26, 50-425 Wroclaw, Poland; 3 Department of Microbiology, Faculty of Pharmaceutical Sciences in Sosnowiec, Medical University of Silesia in Katowice, Jagiellońska 4, 41-200 Sosnowiec, Poland; 4 Specialist Medical Practice—Clinical Periodontology, Żuradzka 15 Street, 32-300 Olkusz, Poland

**Keywords:** blood, platelets, growth factors, in vitro, platelet-rich fibrin, antibiotics, drug resistance, bacteria

## Abstract

The rising prevalence of antibiotic resistance underscores the need for localized drug delivery systems that minimize systemic exposure. Autologous platelet concentrates (APCs), including concentrated platelet-rich fibrin (c-PRF) and liquid-phase concentrated growth factors (LPCGFs), have emerged as potential carriers for antimicrobial agents. This study aimed to evaluate the efficacy of c-PRF and LPCGF as carriers for three antibiotic formulations—amoxicillin with clavulanic acid, clindamycin, and a combination of amoxicillin with metronidazole—against methicillin-sensitive *Staphylococcus aureus* (MSSA), methicillin-resistant *Staphylococcus aureus* (MRSA), and *Enterococcus faecalis* (low-level natural resistance). The disk diffusion method was employed to incorporate antibiotics into both APC types, which were then applied to disks placed on bacterial cultures. The size of the inhibition zones was measured at 20-, 40-, 60-, and 80-h intervals. Every 20 h, the disks were transferred to a new Petri dish. Statistical analysis included Welch’s *t*-test and two-way ANOVA. c-PRF demonstrated superior performance as a carrier for amoxicillin and clindamycin, showing the presence of inhibition zones for up to 60 h. In contrast, LPCGF exhibited greater efficacy when used with the amoxicillin–metronidazole combination, particularly at higher concentrations. Both APCs showed limited effectiveness against *E. faecalis* when combined with clindamycin. The study confirms the suitability of autologous platelet concentrates as localized antibiotic delivery systems. The choice between c-PRF and LPCGF should be guided by the drug’s physicochemical properties and clinical application. APCs offer a promising alternative for targeted antimicrobial therapy in dental and surgical settings.

## 1. Introduction

Antibiotic resistance represents the most pressing global threat to the effective treatment of bacterial infections at present. The impact of antibiotic resistance on clinical and therapeutic outcomes has been well-documented [1]. A broad range of deleterious effects have been linked to antibiotic usage. These adverse effects include hypersensitivity reactions and a variety of dermatological and allergic disorder [2]. Moreover, the unwarranted prescription of antibiotics has the potential to precipitate a range of grave consequences, including bacterial resistance, gastrointestinal disorders, and hematological abnormalities, as well as the diversion of bacterial microbiota. This phenomenon could also contribute to the development of oral bacterial resistance, a matter of growing concern in dentistry and medicine [3]. A substantial body of research has previously demonstrated that approximately 12% of dentists adequately prescribe antibiotics as a prophylactic intervention or treatment [4]. Kelly et al. posited that the direct targeting of tissues with local drug delivery strategies constitutes a viable approach to curtailing the unnecessary use of antimicrobials [5]. Moreover, the delivery of antimicrobials to oral lesions is constrained by systemic circulation. This underscores the significance of drug delivery systems in addressing oral and dental health concerns. These systems consist of transporters and associated therapeutic agents, which are designed to deliver and release targeted compounds or bioactive agents to specific locations within the body at predetermined rates [6].

Recent studies have emerged that have examined the potential for utilizing autologous platelet concentrates (APCs) as carriers for antibiotics [7]. In vivo studies have demonstrated the superiority of antibiotic-loaded platelet-rich fibrin (AL-PRF) over the simultaneous use of platelet-rich fibrin (PRF) and systemic antibiotic administration [8]. In recent years, the use of blood concentrates in dentistry has become increasingly prevalent. The use of autologous blood concentrates in dentistry has been demonstrated to facilitate natural healing, accelerate tissue regeneration, and provide patients with a more comfortable postoperative outcome [9,10]. PRF is arguably the most prominent blood-derived product due to its expeditious and economical preparation method. The process of creating PRF matrices entails a single centrifugation step, resulting in the immediate availability of the product for utilization [11]. In 2014, injectable platelet-rich fibrin (i-PRF) was introduced, developed by modifying centrifugation forces. The development of i-PRF entailed the utilization of reduced centrifugation speeds and the employment of non-glass tubes, resulting in the formation of a flowable form of PRF [12]. In 2020, Miron et al. presented a new method of harvesting liquid concentrated PRF (c-PRF), which allowed platelet and leukocyte counts to increase several-fold [13]. Concentrated growth factors (CGFs) were developed by Sacco in 2006. CGF is produced by centrifugation of blood samples with a special centrifuge device, similar to PRF. However, the different centrifugation speed allows for the isolation of a much larger, denser, and growth factor-rich fibrin matrix [14]. Liquid-phase CGF (LPCGF) preserves the composition of active factors such as transforming growth factor-β (TGF-β) and vascular endothelial growth factor (VEGF), and their biological efficacy, compared to traditional CGF. The presence of CD34+ cells in LPCGF, along with its ability to release growth factors in a sustained manner, contributes to the long-term suppression of inflammation-related factors [15].

The objective of the present study was to ascertain the potential of autologous platelet concentrates as an antibiotic carrier. This investigation sought to determine whether a discrepancy would be observed between APCs and to ascertain the most suitable antibiotic and its optimal concentration for this purpose.

## 2. Results

### 2.1. Clot Formation After Antibiotics Addition

Following the amalgamation of precise quantities of pharmaceuticals with autologous platelet concentrates, the stability of the mixture was evaluated. The evaluation entailed a determination of the mixture’s tendency to clot, as opposed to its tendency to remain liquid. Subsequent to a 15-min period, an evaluation of the substance’s consistency was conducted within a 2 mL Eppendorf tube. For mixtures where lower antibiotic concentrations were used, 0.55 mL of solution was used, while for mixtures where higher antibiotic concentrations were used, 0.625 mL of solution was used. The results demonstrated that only a high concentration of clindamycin (0.3 mg/mL) hindered clot formation, ensuring the mixture remained homogeneous and liquid throughout the experiment. All other concentrations analyzed allowed both PRF and CGF to form a clot. The Eppendorf tubes were placed on a shaker (VORTEX Genius 3 Shaker, Barcelona, Spain) to maintain the liquid state for an extended period, thereby facilitating the continuation of the study.

### 2.2. Antibacterial Effect of APC as Carries for Drugs

#### 2.2.1. Amoxicillin

**Figure 1:**
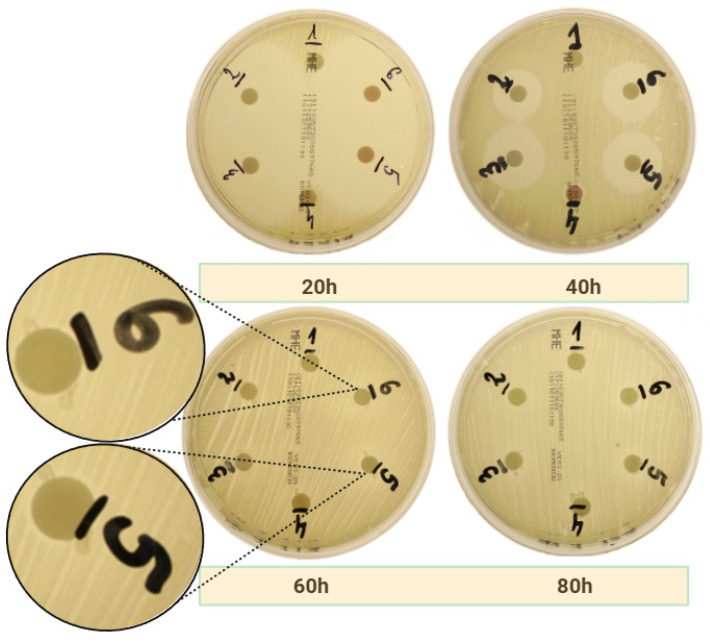
Photographs showing time-dependent zones of inhibition on a strain of *MSSA* bacteria using c-PRF as a carrier for amoxicillin.

As illustrated in Table 1, the results obtained from all groups whose disks contained amoxicillin with clavulanic acid are presented. Statistically significant differences (*p* < 0.05) from their respective control group counterparts are indicated in red. Statistical comparisons were performed against the negative control group using Welch’s *t*-test, with significance considered at *p* ≤ 0.05. The control group that corresponds to group 5 is group 2, and the corresponding group 6 is group 3. Additionally, control group 1 corresponds to test group 4. The substrates of the *S. aureus* ATCC 25923 strain (MSSA) exhibited the largest zones of inhibition, and c-PRF demonstrated significantly superior performance as a carrier. Notably, this carrier demonstrated a zone of inhibition at the 60th hour of the test, which was not observed until the 80th hour in the absence of antibiotic addition. Furthermore, it was observed that at the 40th hour of measurement, both c-PRF and LPCGF exhibited a zone of inhibition in Group 4 (without antibiotic addition). This phenomenon was observed in all plates with the *MSSA* strain, including those with other antibiotics. Furthermore, c-PRF was demonstrated to be a superior carrier for amoxicillin, as evidenced by its efficacy against MRSA and *E. faecalis* bacterial media. Figure 1 illustrates the decrease in zones of inhibition over time in the group treated with c-PRF and amoxicillin on *MSSA* bacterial strain plates.

#### 2.2.2. Clindamycin

**Figure 2:**
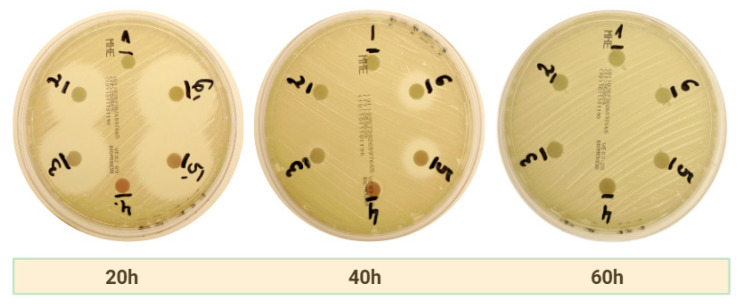
Photographs showing time-dependent zones of inhibition on a strain of *MSSA* bacteria using c-PRF as a carrier for clindamycin.

Table 2 presents the diameter values of the zones of inhibition for the clindamycin groups. Statistically significant values (*p* < 0.05) are indicated in red. Analysis of variance (ANOVA) based on zones of inhibition of all three bacterial strains combined showed that, as carriers of clindamycin, c-PRF showed significantly larger zones of inhibition compared to LPCGF (*p* < 0.05). Consistent with the findings for amoxicillin, c-PRF was identified as a significantly superior carrier.However, a comparison of the extent of inhibition at 40 h in the MSSA strain reveals that LPCGF exhibits a significantly superior outcome. Zones of growth inhibition were observable as early as 20 h after incubation, reaching the highest values in group 6, where the higher drug concentration was employed. In contrast to amoxicillin, zones of inhibition were no longer visible at 60 h of study. Statistically significant differences (*p* < 0.001) with respect to the control group without drug were observed from 20 h onwards for both carriers. For *E. faecalis*, the activity of both carriers was limited, with zones of inhibition remaining within 7 mm, suggesting low sensitivity of this strain to clindamycin in local application. The results of the assay, as shown in Figure 2, indicate a predominant release of antibiotic at the 40-h stage of the assay on a medium containing *MSSA* strains.

#### 2.2.3. Amoxicillin with Metronidazole

**Figure 3:**
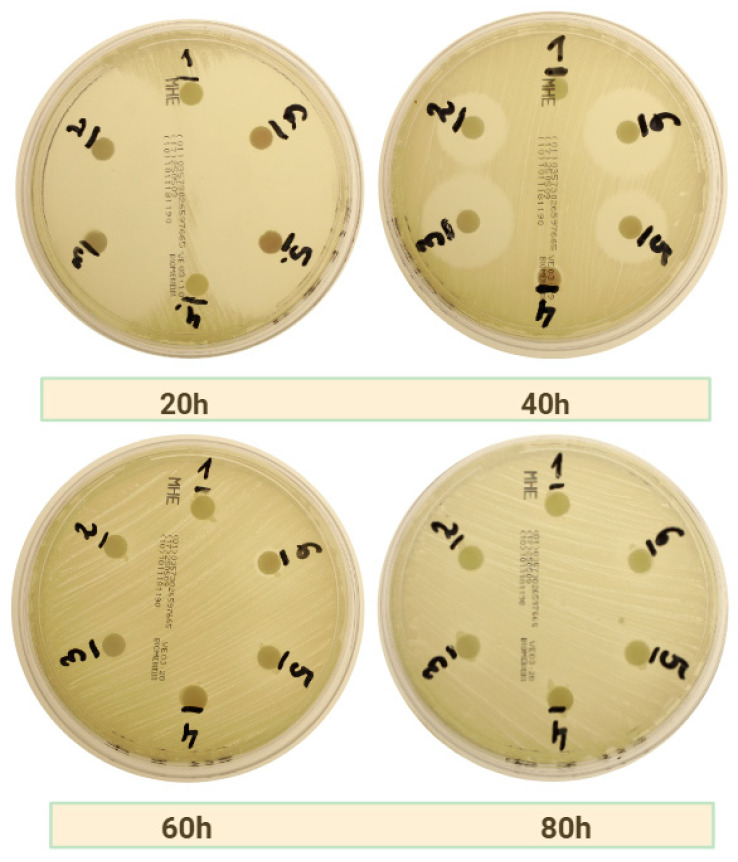
Photographs showing time-dependent zones of inhibition on a strain of *MSSA* bacteria using LPCGFas a carrier for amoxicillin with metronidazole.

As illustrated in Table 3, the diameter values of the zones of inhibition for the groups treated with amoxicillin and metronidazole are presented. Statistically significant values (*p* < 0.05) are highlighted in red. In contrast to the other antibiotics, amoxicillin with metronidazole demonstrated a significantly superior capacity as a carrier, exhibiting its superiority over c-PRF across all bacterial strains examined. As observed with amoxicillin alone, zones of inhibition were also evident for the MSSA strain at 60 h of study. Figure 3 illustrates the decrease in zones of inhibition over time in the group treated with LPCGF and amoxicillin with metronidazole on *MSSA* bacterial strain plates.

## 3. Discussion

### 3.1. General Results

The present study demonstrated that both forms of APCs—namely, c-PRF and LPCGF—effectively served as carriers for antibiotics, contributing to measurable antimicrobial activity against reference bacterial strains. The extent of the inhibition zones was found to be dependent on the type of antibiotic used, the concentration of the drug, the APC formulation, and the bacterial strain tested.

Among the antibiotics evaluated, amoxicillin with clavulanic acid exhibited the most significant zones of inhibition, particularly against MSSA. In the context of the present study, c-PRF demonstrated superior performance in comparison with LPCGF. This observation was characterized by the maintenance of zones of inhibition for a duration of up to 60 h, whereas zones of inhibition with LPCGF exhibited a decline in visibility at an earlier time point. Statistically significant differences in inhibition diameter were observed in both PRF-based and CGF-based groups compared to controls, with the c-PRF + amoxicillin group demonstrating the most prolonged activity. In the case of clindamycin, c-PRF once again exhibited a significant advantage over LPCGF, with the largest zones of inhibition observed at 20 and 40 h. However, after 60 h, a significant reduction in antimicrobial effects was observed, and no inhibition was recorded beyond that point. However, it should be borne in mind that according to Clinical & Laboratory Standards Institute (CLSI) standards, no significant antimicrobial properties can be claimed for zones below 10–12 mm. The efficacy of both carriers was found to be limited against *Enterococcus faecalis*, with inhibition diameters not exceeding much more than 7 mm, thereby indicating a low level of susceptibility for this strain to local clindamycin exposure.

Conversely, the combination of amoxicillin with metronidazole resulted in a reversal of carrier performance, with LPCGF displaying superior activity across all tested strains. This effect was especially prominent at higher concentrations and at the 40-h time point, where the LPCGF-based groups maintained broader inhibition zones than their PRF equivalents. It is noteworthy that both APCs exhibited sustained antimicrobial activity against MSSA, MRSA, and *E. faecalis* for a minimum of 40 h when loaded with the amoxicillin–metronidazole mixture.

The antibiotic concentrations employed in this study were selected with careful consideration of clinical relevance rather than strict alignment with minimum inhibitory concentrations (MICs). While MIC values provide a baseline for antimicrobial efficacy, standardized MICs for the specific combinations of APCs, antibiotics, and bacterial strains tested here are not consistently available in the EUCAST database. Consequently, the present approach centered on the modeling of concentrations based on therapeutic doses that have been commonly reported in both clinical and in vitro studies. This strategy was designed to emulate realistic exposure levels that could be attained in localized drug delivery scenarios, such as alveolar sockets or periodontal defects. Furthermore, cytocompatibility was a critical consideration in the experimental design. The selected concentrations were intentionally maintained below the threshold levels known to induce cytotoxic effects on osteoblasts and fibroblasts, as preserving regenerative potential is imperative when utilizing APCs in surgical and periodontal applications [16,17,18,19,20,21,22]. This balance between therapeutic relevance and biocompatibility reflects the translational intent of our study, while also setting the stage for future investigations into optimized dosing regimens and release kinetics. Current studies on the kinetics of drug release have demonstrated an initial burst release of approximately 80% of the incorporated antibiotic within the first hour, followed by a gradual release over time. The efficacy of this immediate high-concentration exposure in reducing bacterial load has been demonstrated through experimental studies. The sustained release phase has been shown to ensure prolonged antimicrobial activity, which serves to minimize the development of bacterial resistance [7,23].

The augmented antibacterial effectiveness of antibiotics integrated into fibrin matrices—such as PRF—can be ascribed to the structural and biochemical characteristics of fibrin. These characteristics enable the regulated release of drugs and the localized execution of antimicrobial activity. The three-dimensional fibrin network that characterizes PRF functions as a reservoir for antibiotic molecules, thereby enabling sustained drug release as the matrix undergoes gradual degradation [7,23]. The increased potency of antibiotics incorporated into fibrin matrices, such as PRF, can be ascribed to the structural and biochemical characteristics of fibrin. These characteristics facilitate regulated drug release and localized antimicrobial activity. PRF provides a three-dimensional fibrin network that functions as a reservoir for antibiotic molecules, thereby enabling the sustained release of the drug as the matrix gradually degrades. The function of this controlled release mechanism is to prevent the rapid elimination of antibiotics. As a result, the therapeutic drug concentrations at the infection site are maintained for an extended period [7]. Serafini et al. demonstrated continuous release of metronidazole and amoxicillin from A-PRF at concentrations similar to those utilized in our study. Furthermore, the findings demonstrated that metronidazole exhibited a progressively diminished release over time [24]. Furthermore, Ercan et al. demonstrated, based on a study utilizing scanning electron microscopy (SEM), that the addition of an antibiotic (doxycycline) to the PRF clot resulted in enhanced fibrin network strength, consequently leading to a thicker fibrin structure [25]. The primary benefit of PRF is its high concentration of growth factors. It was determined that the lower dose of antibiotics exhibited sustained antimicrobial efficacy, while the release of growth factors from AL-PRF, the structure of platelet–fibrin beams, and the fibrin network remained unaffected [26]. Siawasch et al.’s findings reveal that in the context of AL-PRF, amoxicillin and metronidazole, the antibiotics used in the treatment, do not impede the release of growth factors to a significant extent [27]. Furthermore, a study conducted by Monika et al. demonstrated that platelet concentrates (i-PRF and c-PRF) with metronidazole incorporation can increase cell proliferation to a greater magnitude than i-PRF and C-PRF alone [22].

A multitude of studies have demonstrated that the physical properties of the AL-PRF are largely maintained at reduced antibiotic concentrations. Conversely, elevated concentrations of specific antibiotics, such as metronidazole, have been observed to disrupt PRF formation [27,28]. Notably, even the lowest concentration of vancomycin has been shown to compromise PRF formation [29,30].

Walianto et al. demonstrated that the inhibition of Aggregatibacter actinomycetemcomitans by PRF and I-PRF incorporated with metronidazole was greater compared to PRF and I-PRF without incorporating metronidazole. Furthermore, the inhibition of A. actinomycetemcomitans by I-PRF with metronidazole incorporated was greater than that by PRF with metronidazole incorporated [31].

As demonstrated by Straub et al., the selection of centrifugation protocol exerts a substantial influence on the characteristics of APC as a carrier for antibiotics. A series of experiments were conducted to assess the efficacy of three distinct protocols: protocol A (1300 rpm, 8 min, RCF-max = 208 g), protocol B (2300 rpm, 12 min, RCF-max = 652 g), and protocol C (1500 rpm, 14 min, RCF-max = 276 g). The results of these experiments indicated that protocol B yielded the most substantial inhibition zone [32]. The present study employed a protocol to obtain c-PRF, the settings of which are closest to protocol B from the aforementioned study, due to the use of high RCF values.

Overall, c-PRF was determined to be a more suitable carrier for individual antibiotics such as amoxicillin with clavulanic acid and clindamycin, likely due to its more porous and open fibrin matrix, which facilitates diffusion and early release of hydrophilic drugs. In contrast, LPCGF appeared to be better suited for the more complex dual antibiotic formulation of amoxicillin with metronidazole, possibly owing to its denser fibrin matrix and greater retention capacity, allowing for more sustained drug release [33]. A study employing SEM has demonstrated that CGF exhibits a significantly more compact cross-linking of fibrin fibers in comparison to PRF [34]. The hypothesis that the alternated speeds of CGF may influence the agglutination of platelets, fibrinogen, factor XIII, and thrombin, which facilitate the conversion of fibrinogen to fibrin and the polymerization of the fibrin, is also supported by these findings [35,36]. To the authors’ knowledge, this is the first study to test the properties of CGFs as carriers for antibiotics.Findings of this study underscore the potential for APC selection tailored to drug characteristics and therapeutic goals.

The authors of the present study published a systematic review on this topic prior to designing the methodology, in which they described in detail the differences in the various methods for creating AL-PRF [7]. Consequently, the decision was made to administer antibiotics directly into the liquid phase of the APC. This resulted in a homogeneous distribution of the antibiotic with a known concentration. A majority of the studies that have focused on autologous platelet-rich fibrin (AL-PRF) have employed either direct injection into the PRF clot or blood collection from the patient undergoing antibiotic therapy. With injection into the PRF clot, homogeneous distribution of the antibiotic cannot be achieved. In the case of when antibiotics are initially administered intravenously and blood is collected, the capacity of APC loading can be measured in comparison to plasma concentration. In such cases, it is imperative to collect blood samples at the optimal time to achieve clinically significant concentrations in APCs. Typically, this involves the estimation of the peak plasma concentration of the antibiotic [37]. For a considerable number of intravenously administered antibiotics, this occurrence may transpire towards the conclusion of the infusion or in the immediate aftermath [38].

### 3.2. Limitations

When interpreting the results of this study, it is essential to consider its numerous limitations. Firstly, the study is in vitroand therefore does not reflect conditions that occur in the invivo environment. Such conditions include tissue perfusion, immune system interactions, enzymatic degradation, and local pH. These factors may significantly alter the drug release kinetics and antimicrobial efficacy of APC-based delivery systems in clinical settings. Furthermore, the utilization of reference strains in lieu of wild-type strains isolated from patients is a notable shortcoming that must be addressed in future studies to ensure the relevance and generalizability of the findings. A significant limitation of the study is the absence of analysis of drug release kinetics.The study’s primary focus on inhibition zone measurements, which reflect antimicrobial activity, does not provide quantitative data on drug concentration or release kinetics. Absent techniques such as HPLC or ELISA, the precise dynamics of drug liberation from PRF and CGF remain speculative. While APCs demonstrated efficacy, a comparative analysis against standard local delivery materials (e.g., hydrogels, collagen sponges, or slow-release microspheres) is necessary to provide a more comprehensive contextual framework for evaluating their efficacy and potential advantages or disadvantages. It is also noteworthy to consider that the carriers were applied to cellulose disks, which may have interfered with fibrin network formation in the APC. While the results indicate the superiority of the carriers, the authors hypothesize that the absence of the disks could potentially enhance the efficiency of drug release. Despite this, it was not decided to use the well-diffusion method because, with this method, there is no possibility of transferring the corresponding substance from the wells to the new substrate after 20 h.

### 3.3. Future Implications

#### 3.3.1. Osteonecrosis of the Jaw

Osteonecrosis of the jaw is a condition in which bone cells undergo necrosis due to various etiologies. The condition is classified into distinct categories, namely drug-induced jaw osteonecrosis, osteoradionecrosis, traumatic osteonecrosis, non-traumatic osteonecrosis, and spontaneous osteonecrosis. Antiresorptive or antiangiogenic pharmaceutical agents have been associated with the development of drug-induced osteonecrosis. The combination of medications, microbial contamination, and local trauma has been identified as the underlying cause of this condition [39,40]. In patients diagnosed with osteonecrosis of the jaws (ONJ), systemic targeted antibiotic therapy for necrotic lesions may demonstrate reduced efficacy in comparison to its effectiveness for other indications. This phenomenon can be attributed to the inadequate blood supply characteristic of necrotic lesions within the jaws. Consequently, this results in a diminished distribution of the systemically administered antibiotic [41,42,43]. Al-PRF has the potential to be utilized in the delivery of antibiotics to sites with necrotic lesions. This is due to two factors: first, the continuous release of antibiotics, and second, the utilization of PRF for the prevention and treatment of ONJ lesions [44,45].

#### 3.3.2. Periodontitis and Periimplantitis

Periodontitis is defined as an inflammatory disease of the supporting structures of teeth. It is of microbial origin and is modified by multiple host and environmental factors [46,47]. A recent systematic review by Niemczyk et al. demonstrated that the utilization of i-PRF and PRP in the non-surgical management of periodontitis results in a substantial enhancement in clinical parameters when compared to SRP alone [48]. Thamaraiselvan and Jayakumar, in their randomized trial, demonstrated that the use of i-PRF as a vehicle for ciprofloxacin in the non-surgical treatment of periodontitis resulted in a significant improvement in clinical parameters [49]. Metronidazole is among the most prevalent antibiotics utilized in the treatment of periodontal disease [50]. Concurrently, a meta-analysis by Sgolaster et al. demonstrated that the incorporation of metronidazole during SRP significantly enhances treatment outcomes [51]. A systematic review by Zandbergen et al. on the use of the combination of metronidazole and amoxicillin in the treatment of periodontitis showed similar results [52]. Miani PK et al. concluded that the experimental gel group, which was based on 15% metronidazole, was superior to the control group in terms of reducing bacterial counts after the intervention [53].

In the treatment of peri-implantitis and periodontal diseases, antimicrobials such as nitroimidazole, doxycycline, and beta-lactam antibiotics have been administered systemically [54]. The use of topical antibiotic carriers, such as c-PRF and LPCGF, holds promise in enhancing the quality of treatment for patients with periodontitis and peri-implant tissue in the future.

#### 3.3.3. Tooth Extraction

Tooth extraction is the most common procedure in the field of oral surgery [55]. There are many recommendations and publications relating to the lack of need for antibiotics in extractions (for example, of lower third retained molars) when there is no accompanying inflammation and when the patient is healthy [56,57,58]. Nevertheless, a large proportion of clinicians choose to implement antibiotic therapy because of the significant reduction in the risk of dry socket and the reduction in swelling and inflammation, along with pain [59,60,61]. Due to the beneficial impact of antibiotics on patient recovery, a significant proportion of medical professionals opt to disregard the recommendation [62]. Donmezer and Bilginaylar’s study demonstrated that the outcomes of local and systemic antibiotic administration with the use of PRF after mandibular third molar surgery were analogous, with both exhibiting a statistically significant reduction in pain and analgesic intake. The implementation of these procedures resulted in a reduction of trismus and swelling when compared to the control group [8]. The utilization of AL-PRF has the potential to facilitate the continuous release of antibiotics at the extraction site, thereby significantly mitigating the escalation of antibiotic resistance.

#### 3.3.4. Sticky Bone

A new concept of producing a growth factor-enriched bone graft matrix, also known as “sticky bone”, using autologous fibrin-rich blocks with CGF was first demonstrated by Sohn and colleagues in 2011 [63] and later by Mourão et al. [64] with liquid/injectable platelet-rich fibrin (i-PRF) matrices [65]. Some clinicians recommend the addition of antibiotics to sticky-bone preparations based on a combination of hard tissue graft and autologous platelet concentrates. The incorporation of antibiotics into the fibrin structure may facilitate enhanced kinetics of augmented drug release. The use of low doses of clindamycin and amoxicillin for intraoral bone graft decontamination is supported by data, while concerns have been raised about the use of chlorhexidine (CHX). The use of low doses of clindamycin has been shown to favor osteoblast growth and differentiation, positioning it as a promising decontaminant for intraoral bone grafts. In contrast, CHX has been identified as a less bone-friendly agent [34].

## 4. Materials and Methods

### 4.1. Population and Study Design

The study was approved by the Bioethics Committee of the Silesian Medical University in Katowice on 18 February 2025. The committee’s decision was assigned the number BNW/NWN/0052/KB1/10/25. The study was conducted in accordance with the European Committee on Antimicrobial Susceptibility Testing (EUCAST) guidelines, employing the disk-diffusion method. The inclusion and exclusion criteria, delineated in Table 4, guided the selection of study participants. A total of 10 team members and volunteers provided blood samples for the study.

### 4.2. APC Preparation

The Biomedico 21G (0.8 mm) butterfly needles, with a length of 19 mm, were utilized for the collection of blood. CDRICH plastic tubes, devoid of additives, with a capacity of 9 mL and dimensions of 16 × 100 mm, were utilized to obtain concentrated liquid PRF for injection (c-PRF) and liquid-phase CGF (LPCGF). A water test was performed to confirm the absence of additional chemicals in the tubes. For the injection of c-PRF, the iFugeC4000 NXT centrifuge (Neuation Technologies, Wecker, Grevenmacher, Luxembourg), operating horizontally, was employed. The centrifuge settings for c-PRF were set to 8 min, with a maximum relative centrifugal force (RCF) of 2000× *g*. For LPCGF, the Medifuge MF200 centrifuge (Silfradent Srl, Forlì, Italy) was utilized. The protocol for obtaining LPCGF involved a sequence of four distinct protocols: 30 s acceleration, 2 min × 2700 rpm, 4 min × 2400 rpm, 4 min × 2700 rpm, 3 min × 3000 rpm, and 36 s deceleration, stop.

Subsequently, both c-PRF and LPCGF were meticulously extracted from the tubes using a syringe and needle, with the needle being strategically positioned in the buffy coat zone at the border with the red blood cell layer. Subsequently, a volume of 0.5 milliliters of platelet concentrate was extracted from each tube.

### 4.3. AL-PRF Preparation

The solutions of amoxicillin with clavulanic acid, clindamycin, and amoxicillin with clavulanic acid dissolved in metronidazole were added to APCs obtained by centrifugation. The precise quantities of the antibiotic agents were meticulously measured using a sterile automatic pipette. The prepared solutions were then transferred to a 2mL Eppendorf tube for further processing. Following this step, 20 µL of each preparation was collected and applied to a sterile disk (BLANK DISCS, Oxoid Ltd., Basingstoke, UK) with a diameter of 6 mm.

### 4.4. Study Groups

The study encompassed twelve distinct study groups, as delineated in Table 5. Each group was subjected to three replicates in separate Petri dishes. Each dish contained all groups for a given APC and bacterial strain. To mitigate individual differences, each Petri dish contained groups of APCs centrifugally derived from the same individual. Each repetition of the same dish layout contained blood products from other study participants.

### 4.5. Bacterial Strains

The following table (Table 6) illustrates the bacterial strains that were meticulously selected for utilization in the present study, along with the designated medium for each.

**Figure 4:**
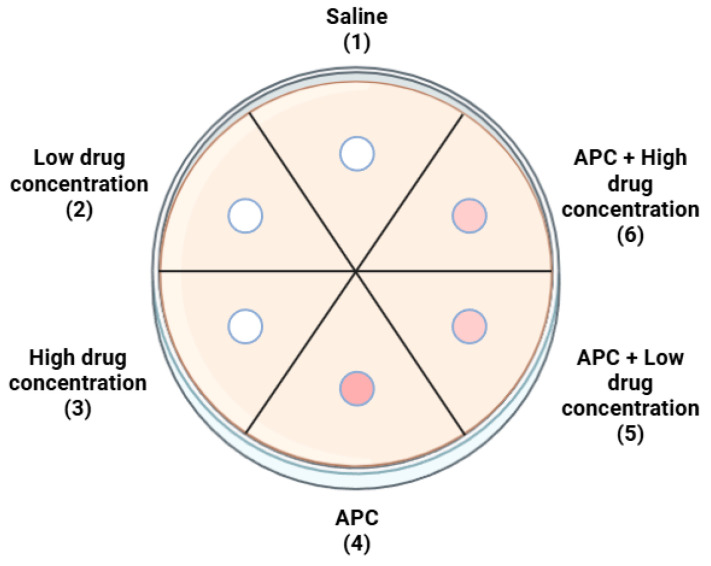
Arrangement of groups on the dishes.

The sequence of the dishes remained constant. The following figure illustrates the distribution of groups on the dish (Figure 4).

### 4.6. Microbiological Analysis

Each strain was cultivated in a 90mmdiameter Petri dish containing agar (bioMerieux, Marcy-l’Étoile, France), with a depth of 4 mm (approximately 25 mL). The surface of the agar remained dry and homogeneous. Following a 24-h incubation period at 37 °C and under aerobic conditions, the colony sample was carefully removed from the surface of the plate and suspended in a sterile saline solution (0.9% NaCl). The number of viable cells in the suspension was determined by measuring the optical density at 525 nm using a Densi-La-Meter II densitometer (ERBA LACHEMA, Prague, Czech Republic). The McFarland scale was used to determine the optical density, with a value of 0.5 corresponding to approximately 1.2 × 10^8^ CFU/mL.

Subsequently, 15 min following the preparation, a sterile cotton swab was immersed in the suspension. The plates were then inoculated manually by spreading the suspension in three directions onto the surface of Mueller–Hinton agar (bioMerieux, Marcy-l’Étoile, France) using the swab (for each strain separately). Disks with specific APCs were applied within 15 min of inoculation.

Subsequent to each photography session (every 20 h), the disks were transferred to a new Petri dish with the inoculums applied, and the process was repeated until no zones of inhibition greater than 6 mm were obtained throughout the dish.

Cultures were then subjected to incubation at a temperature of 35 ± 2 °C under conditions of aerobic growth in a MIR-262 SANYO laboratory incubator (Etten-Leur, The Netherlands). The extent of the inhibition zone was reported as the primary outcome measure.

### 4.7. Measurement of Inhibition Zones

Following 20, 40, 60, and 80 h of incubation, the Petri dishes were extracted from the incubator and imaged using a Panasonic DMC-G80 Lumix camera (Osaka, Japan) equipped with a Lumix Panasonic H-FS 2060 12-60 micro-HD lens (Osaka, Japan) from a constant distance of 30 cm at 90 degrees to the surface. The camera was mounted on a tripod. All photographs were taken under identical conditions.

The diameter of the inhibition zones surrounding the individual disks was subsequently measured after the completion of all photographic sessions. The analysis was carried out using the ImageJ—Fiji software platform version 1.53j (US National Institutes of Health, Bethesda, MD, USA) (Date of last measurement: 11 April 2025). The results are reported in millimeters (mm).

Prior to measurement, the size of the microbial inhibition zone was calibrated from left to right along the boundary parallel to the base of the image, ensuring that the line passes through the center of the diffusion disk. The results obtained were then recorded and subsequently subjected to statistical analysis.

### 4.8. Statistical Analysis

The results were presented as mean and standard deviation (M ± SD). Due to the limited sample size (n = 3 in each group) and the potential inequality of variances, a Student’s *t*-test version of independent samples with unequal variances, known as the Welch test, was employed. The alternative hypothesis was formulated unilaterally, assuming that the use of a carrier leads to an increase in the inhibition zone. A two-factor analysis of variance (two-way ANOVA) with replication was employed to assess the effect of antibiotic concentration and the presence of the carrier on the size of the zone of inhibition. This analysis enabled the assessment of the primary effects of the two factors, as well as their potential interaction. A *p*-value ≤ 0.05 was considered to indicate a statistically significant difference. The statistical analysis was performed using Statistica, version 7.1 PL (StatSoft Poland, Krakow, Poland).

## 5. Conclusions

This in vitro study demonstrated that both c-PRF and LPCGF can serve as effective carriers for selected antibiotics, although their performance varies depending on the drug and bacterial strain. c-PRF showed superior antibacterial activity when used with amoxicillin with clavulanic acid and clindamycin, particularly against MSSA, with inhibition zones maintained up to 60 h. In these groups, c-PRF exhibited larger and more sustained zones of inhibition compared to LPCGF. In contrast, LPCGF showed slightly greater efficacy as a carrier for the combination of amoxicillin with metronidazole, especially at higher concentrations and in later time points, suggesting a more favorable release profile for complex antibiotic formulations. Antimicrobial activity against MRSA was generally effective across all formulations, with both APCs maintaining comparable inhibition. However, both c-PRF and LPCGF demonstrated limited efficacy against Enterococcus faecalis when combined with clindamycin. These findings suggest that the selection of an APC type as a drug carrier should consider the chemical nature of the incorporated antibiotic and its release profile requirements. The study provides experimental evidence supporting the potential for APCs to be used in localized antibacterial therapy, particularly in the context of infections caused by MSSA and MRSA.

## Figures and Tables

**Table 1:** Mean diameters of zones of inhibition with standard deviation for the amoxicillin groups.

Bacteria Strain	APC	Time of Measurement	Group 1	Group 2	Group 3	Group 4	Group 5	Group 6
*S. aureus* (MSSA)	c-PRF	20 h	-	41.07 (±0.13)	43.86 (±0.10)	-	41.86 (±0.41)	44.25 (±0.08) *
		40 h	-	16.55 (±0.64)	21.76 (±0.10)	7.16 (±0.03) *	20.51 (±0.69) *	22.58 (±0.38)
		60 h	-	-	-	-	6.97 (±0.03) *	7.02 (±0.04) *
		80 h	-	-	-	-	-	-
	LPCGF	20 h	-	40.32 (±0.48)	44.67 (±0.39)	-	41.24 (±0.43)	45.51 (±0.46)
		40 h	-	17.52 (±0.38)	22.33 (±0.11)	7.21 (±0.11)	17.86 (±0.32)	22.85 (±0.34)
		60 h	-	-	-	-	-	-
MRSA	c-PRF	20 h	-	16.27 (±0.13)	20.46 (±0.46)	-	17.10 (±0.42)	23.43 (±0.29) *
		40 h	-	-	-	-	-	-
	LPCGF	20 h	-	20.16 (±0.17)	23.01 (±0.4)	-	22.03 (±0.15) *	23.44 (±0.24)
		40 h	-	-	-	-	-	-
*E. faecalis*	c-PRF	20 h	-	32.02 (±0.10)	35.74 (±0.10)	-	34.08 (±0.17) *	36.29 (±0.16) *
		40 h	-	15.96 (±0.07)	20.36 (±0.11)	-	17.39 (±0.12) *	20.72 (±0.11)
		60 h	-	-	-	-	-	-
	LPCGF	20 h	-	36.04 (±0.16)	36.21 (±0.22)	-	36.13 (±0.16)	20.97 (±0.58)
		40 h	-	17.60 (±0.28)	20.34 (±0.19)	-	18.75 (±0.47)	20.48 (±0.29)
		60 h	-	-	-	-	-	-

APC—autologous platelet concentrate;c-PRF—concentrated platelet-rich fibrin; LPCGF—liquid-phase concentrated growth factor;MSSA—methicillin-sensitive *Staphylococcus aureus*; MRSA—methicillin-resistant *Staphylococcus aureus*; “-” marks values where the radius of the inhibition zone was 6 mm. Values shown are in mm and represent the diameters of the inhibition zone. In red are statistically significant values (*p* < 0.05) based on Welch’s *t*-test of corresponding groups with the same drug concentration; * indicates *p*-value < 0.001.

**Table 2:** Mean diameters of zones of inhibition with standard deviation for the clindamycin groups.

Bacteria Strain	APC	Time of Measurement	Group 1	Group 2	Group 3	Group 4	Group 5	Group 6
*S. aureus* *(MSSA)*	c-PRF	20 h	-	24 (±0.08)	28.96 (±0.06)	-	30.38 (±0.43) *	32.15 (±0.07) *
		40 h	-	-	-	7.07 (±0.02) *	12.96 (±0.08) *	13.05 (±0.32) *
		60 h	-	-	-	-	-	-
	LPCGF	20 h	-	24.26 (±0.20)	28.54 (±0.49)	-	30.19 (±0.14) *	31.18 (±0.15)
		40 h	-	-	-	7.97 (±0.34) *	13.79 (±0.29) *	14.46 (±0.46) *
		60 h	-	-	-	-	-	-
*MRSA*	c-PRF	20 h	-	15.32 (±0.31)	20.66 (±0.15)	-	21.38 (±0.36) *	21.40 (±0.40)
		40 h	-	-	-	-	-	-
	LPCGF	20 h	-	16.67 (±0.75)	20.65 (±0.24)	-	19.38 (±0.59)	20.97 (±0.58)
		40 h	-	-	-	-	-	-
*E. faecalis*	c-PRF	20 h	-	7.29 (±0.33)	7.26 (±0.05)	-	7.32 (±0.32)	7.26 (±0.06)
		40 h	-	-	-	-	-	-
	LPCGF	20 h	-	6.56 (±0.31)	6.96 (±0.13)	-	7.04 (±0.04)	7.12 (±0.02)
		40 h	-	-	-	-	-	-

APC—autologous platelet concentrate;c-PRF—concentrated platelet-rich fibrin; LPCGF—liquid-phase concentrated growth factor; MSSA—methicillin-sensitive *Staphylococcus aureus*; MRSA—methicillin-resistant *Staphylococcus aureus*; “-” marks values where the radius of the inhibition zone was 6 mm. Values shown are in mm and represent the diameters of the inhibition zone. In red are statistically significant values (*p* < 0.05) based on Welch’s *t*-test of corresponding groups with the same drug concentration; * indicates *p*-value < 0.001.

**Table 3:** Mean diameters of zones of inhibition with standard deviation for the amoxicillin-with-metronidazole groups.

Bacteria Strain	APC	Time of Measurement	Group 1	Group 2	Group 3	Group 4	Group 5	Group 6
*S. aureus* (MSSA)	c-PRF	20 h	-	41.50 (±0.15)	45.77 (±0.04)	-	41.90 (±0.12)	46.11 (±0.12)
		40 h	-	19.05 (±0.27)	24.04 (±0.07)	8.07 (±0.18) *	19.66 (±0.12)	24.11 (±0.21)
		60 h	-	-	-	-	6.40 (±0.70)	7.16 (±0.02) *
		80 h	-	-	-	-	-	-
	LPCGF	20 h	-	41.13 (±0.15)	44.59 (±0.33)	-	41.42 (±0.05)	46.24 (±0.10)
		40 h	-	18.98 (±0.15)	23.33 (±0.21)	7.05 (±0.15)	19.43 (±0.25)	25.02 (±0.34)
		60 h	-	-	-	-	-	7.05 (±0.11)
		80 h	-	-	-	-	-	-
MRSA	c-PRF	20 h	-	17.05 (±0.07)	21.01 (±0.13)	-	17.09 (±0.05)	21.09 (±0.11)
		40 h	-	-	-	-	-	-
	LPCGF	20 h	-	16.81 (±0.28)	21.01 (±0.20)	-	17.42 (±0.26)	22.25 (±0.74)
		40 h	-	-	-	-	-	-
*E. faecalis*	c-PRF	20 h	-	31.89 (±0.32)	33.37 (±0.23)	-	32.14 (±0.19)	33.41 (±0.50)
		40 h	-	15.00 (±0.22)	19.73 (±0.35)	-	15.41 (±0.42)	19.92 (±0.26)
		60 h	-	-	-	-	-	-
	LPCGF	20 h	-	31.96 (±0.23)	34.17 (±0.59)	-	32.01 (±0.23)	35.00 (±0.80)
		40 h	-	14.95 (±0.25)	19.69 (±0.41)	-	15.12 (±0.53)	20.82 (±0.38)
		60 h	-	-	-	-	-	-

APC—autologous platelet concentrate; c-PRF—concentrated platelet-rich fibrin; LPCGF—liquid-phase concentrated growth factor; MSSA—methicillin-sensitive *Staphylococcus aureus*; MRSA—methicillin-resistant *Staphylococcus aureus*; “-” marks values where the radius of the inhibition zone was 6 mm. Values shown are in mm and represent the diameters of the inhibition zone. In red are statistically significant values (*p* < 0.05) based on Welch’s *t*-test of corresponding groups with the same drug concentration; * indicates *p*-value < 0.001.

**Table 4:** Inclusion and exclusion criteria.

Inclusion Criteria:	Exclusion Criteria:
- At least 18 years of age - No systemic illnesses - A platelet count of more than 200,000 in 1 mm^3^ of blood (blood tests performed a maximum of 48 h prior to blood collection for the medical experiment) - Voluntary consent for collection of 30 mL of blood	- History of systemic antifungal treatment within the last 6 months - Pregnant women - Alcohol consumption within the last 7 days - Lactation - Ingested vaccine within the last 3 months - Ongoing menstruation - Allergy to at least one of the agents used in the study - Taking NSAIDs within the last 8 weeks

NSAIDs—non-steroidal anti-inflammatory drugs.

**Table 5:** Study groups and their exact drug concentrations.

Drug Incorporated into APC	Amount of Drug Added per mL of APC (c-PRF/LPCGF)	Concentration of Drug in APC
Amoxicillin with clavulanic acid (Amoksiklav^®^ 500 mg + 100 mg)	0.1 mL (5 mg + 1 mg)	0.05 mg/mL
	0.25 mL (12.5 mg + 2.5 mg)	0.12 mg/mL
Clindamycin (Clindamycin-MIP^®^ 150 mg/mL)	0.1 mL (15 mg)	0.14 mg/mL
	0.25 mL (37.5 mg)	0.3 mg/mL
Amoxicillin with clavulanic acid + Metronidazole (Amoksiklav^®^ 500 mg + 100 mg + Metronidazole Braun^®^ 5 mg/mL)	0.1 mL (5 mg + 1 mg) AMX + 0.5 mg MET	0.05 mg/mL AMX + 0.003 mg/mL MET
	0.25 mL (12.5 mg + 2.5 mg) AMX + 1.25 mg MET	0.12 mg/mL AMX + 0.01 mg/mL MET

AMX—amoxicillin; MET—metronidazole.

**Table 6:** The reference strains used and the media for their culture.

Bacterial Strain	Culture Media	ATCC Number
*Staphylococcus aureus* MRSA	Mueller–Hinton agar	43300
*Staphylococcus aureus* MSSA	Mueller–Hinton agar	25923
*Enterococcus faecalis*	Mueller–Hinton agar	29212

ATCC—American Type Culture Collection; MSSA—methicillin-sensitive *Staphylococcus aureus*; MRSA—methicillin-resistant *Staphylococcus aureus*.

## Data Availability

The data presented in this study are available upon request made to the corresponding author.
